# Circulating tumour-derived DNA in metastatic soft tissue sarcoma

**DOI:** 10.18632/oncotarget.24278

**Published:** 2018-01-19

**Authors:** Nicholas C. Eastley, Barbara Ottolini, Rita Neumann, Jin-Li Luo, Robert K. Hastings, Imran Khan, David A. Moore, Claire P. Esler, Jacqueline A. Shaw, Nicola J. Royle, Robert U. Ashford

**Affiliations:** ^1^ University Hospitals of Leicester NHS Trust, Trauma and Orthopaedics, Leicester, UK; ^2^ Nottingham University Hospitals NHS Trust, Nottingham, UK; ^3^ University of Leicester Department of Genetics, Leicester, UK; ^4^ University of Leicester Department of Cancer Studies, Leicester, UK

**Keywords:** soft tissue sarcoma, biomarker, circulating tumour DNA, cell free DNA, telomere

## Abstract

Following treatment 40% of soft tissue sarcoma (STS) patients suffer disease recurrence. In certain cancers circulating cell free DNA (cfDNA) and circulating tumour-derived DNA (ctDNA) characteristics correlate closely with disease burden, making them exciting potential sources of biomarkers. Despite this, the circulating nucleic acid characteristics of only 2 STS patients have been reported to date.

To address this we used an Ion AmpliSeq™ panel custom specifically designed for STS patients to conduct a genetic characterisation of plasma cfDNA, buffy coat (germline) DNA and where available Formalin-Fixed Paraffin-Embedded (FFPE) primary STS tissue DNA in a cohort of 11 metastatic STS patients. We found that total cfDNA levels were significantly elevated in the STS patients analysed, and weakly correlated with disease burden. Using our Ion AmpliSeq™ panel we also successfully detected ctDNA in 4/11 (36%) patients analysed with a wide variety of STS subtypes and disease burdens. This evidence included the presence of cancer associated *TP53* / *PIK3CA* mutations in 2 patients’ plasma and matched primary STS tumour tissue, and in the plasma alone for 2 patients. We also identified 2 potential examples of allelic loss of heterozygosity in an additional patient's STS DNA and cfDNA.

This is the largest study performed characterising STS patient cfDNA/ctDNA and confirms that the field remains an attractive potential source of novel STS biomarkers. Further work is required to investigate the circulating nucleic acid characteristics of individual STS subtypes, and the potential prognostic or therapeutic roles that cfDNA/ctDNA may hold for patients with these complex tumours.

## INTRODUCTION

Soft tissue sarcomas (STSs) are a heterogeneous group of malignant solid tumors derived from mesenchymal origin. Presently the curative treatment of STSs revolves around surgical resection and peri-operative radiotherapy [1]. Unfortunately following this treatment the aggressive biological behaviour of many STS subtypes means that 17%-24% of tumors will either recur locally [2] or with metastatic disease [3]. Although this recurrence is a difficult problem to manage, those patients with isolated local recurrence and/or oligometastatic disease may be treated curatively with a radical approach that may include surgery, stereotactic radiotherapy and/or radiofrequency ablation. As a result, recurrent STS patients’ prognoses is heavily dependent on the volume of their recurrent disease, making early diagnosis highly desirable [4].

At present no reliable circulating biomarkers exist for STS. An unfortunate consequence of this is that STS recurrence is often multifocal and/or extensive when diagnosed, leaving the majority of patients with palliative treatment options alone. The presence of small DNA fragments freely circulating in the blood stream as a result of different physiological mechanisms of cell death is well established and referred to as cell free DNA or cfDNA [5]. The high turnover rate and necrosis of malignant cells compared with healthy cells means that in cancer patients a high proportion of cfDNA is released from tumour cells within primary and/or metastatic lesions (termed circulating tumour-derived DNA or ctDNA) [5]. In several malignant tumours, ctDNA characteristics correlate closely with tumour burden, disease recurrence and treatment resistance, highlighting ctDNA as an exciting potential source of biomarkers in cancer patients [6–9]. To date only two single case studies [10, 11] have evaluated the presence (and so the potential clinical relevance) of cfDNA/ctDNA in STS patients. As a consequence very little is known about the circulating nucleic acid characteristics of STS patients, including what proportion of STSs shed DNA into the circulation. To address this paucity of knowledge we aimed to characterise the cfDNA levels of a cohort of 11 STS patients with metastatic disease, and using targeted next generation sequencing (tNGS) also investigate the same patients’ ctDNA characteristics.

## RESULTS

### Patient and tumour characteristics

The 11 patients (5F:6M) enrolled for analysis had a mean age of 68.8 years (range 52.2-84.9) and a range of STS subtypes (see Table 1). The patients’ mean RECIST 1.1 score was 161 (25.2-341.9). Appropriate serial radiological investigations enabled disease state to be calculated in seven patients at enrolment. Of these, six had radiological progressive disease and one had stable disease. None of the patients analysed received any systemic oncological treatment or radiotherapy prior to sample collection.

**Table 1:** Metastatic STS patient demographics, clinical and cfDNA characteristics

Participant number	Sex	Age	Histological subtype	STS Trojani grade	cfDNA concentration (ng/ml plasma)	Disease burden (RECIST 1.1 Score)	Disease state	Evidence ctDNA	Overall survival (months)	Date FFPE STS tissue collection	Date cfDNA collection
1	F	74	Leiomyosarcoma	1	105.9	329	-	Y	5.6	16/01/2015	09/03/2015
2	F	79	Leiomyosarcoma	2	26.6	70	PD	Y	n/a	12/06/2014	09/03/2015
3	M	46	Undifferentiated pleomorphic sarcoma	3	62.6	194	-	Y	11.5	20/01/2016	29/06/2015
4	M	86	Soft Tissue Chondrosarcoma	3	9.0	44	PD	Y	n/a	03/10/2013	14/09/2015
5	M	83	Epithelioid Angiosarcoma	3	89.4	98	-	Y	n/a	18/11/2015	30/11/2015
6	F	73	Undifferentiated pleomorphic sarcoma	3	90.7	218	PD	N	2.8		
7	M	52	Synovial sarcoma	3	37.6	187	-	N	14.9		
8	F	72	Extra-skeletal myxoid chondrosarcoma	2	40.6	97	PD	N	n/a		
9	M	47	Spindle Cell Sarcoma	-	38.1	342	PD	N	5.9		
10	M	80	Liposarcoma	-	10.5	164	PD	N	4.9		
11	F	57	Synovial sarcoma	3	21.1	25	SD	N	n/a		

RECIST 1.1 scores represent the sum diameter of all measureable lesions (mm). Disease burden and states (where serial comparable imaging was available) were calculated according to RECIST 1.1 criteria. PD – progressive disease, SD – stable disease. Overall survival is shown in months for those patients that died during the period of analysis.

### Cell free DNA concentration

**Figure 1:**
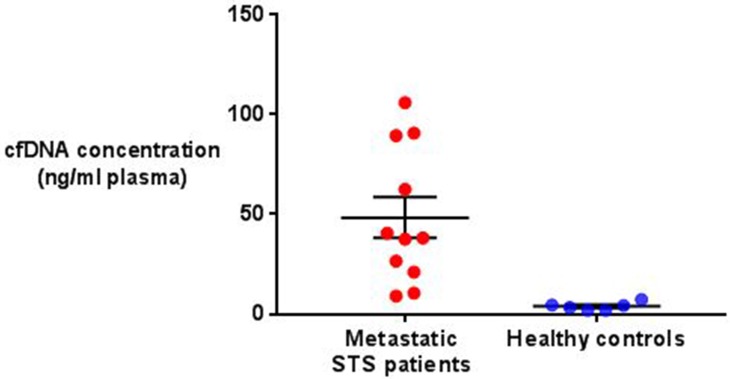
Comparison of total cfDNA concentration in metastatic STS patients and healthy controls The mean and standard error of the mean are represented by horizontal bars. Mean STS patient cfDNA concentration was 48.37ng/ml (± 10.21 SEM, n=11) compared with 3.9 ng/ml (± 0.8 SEM, n=6) in the healthy controls. This difference was significant (P=0.006, unpaired *t*-test).

**Figure 2:**
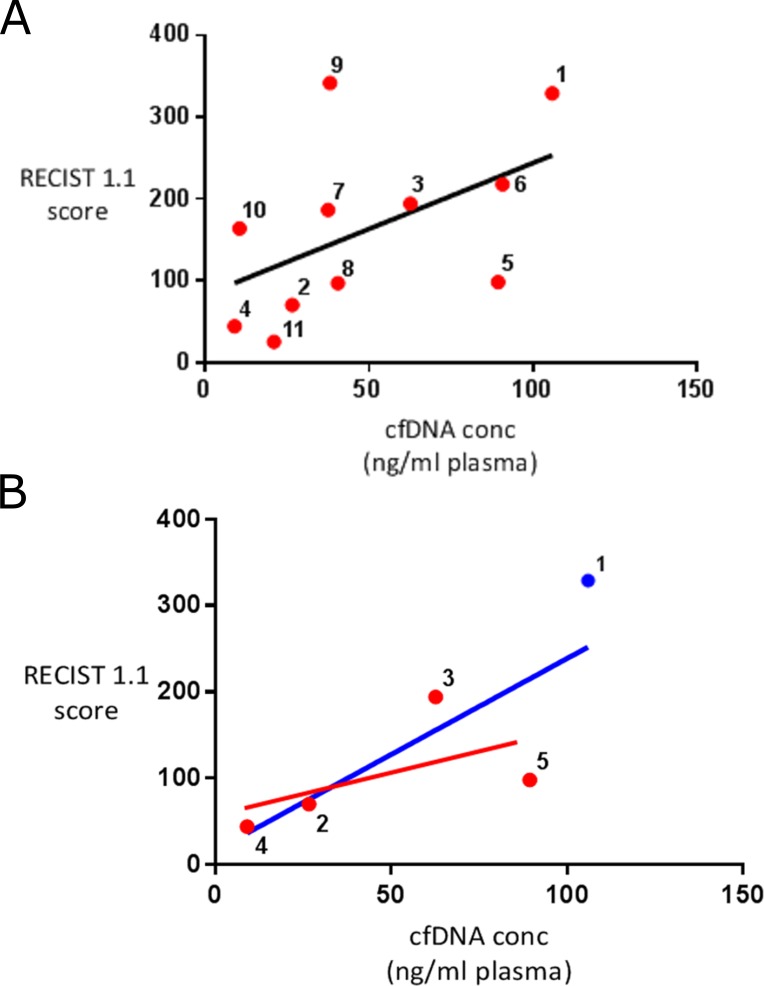
Correlation between disease burden and cfDNA concentration in STS patients **(A)** includes all of the STS patients analysed showing a weak positive linear relationship between disease burden and cfDNA concentration (R^2^=0.26). **(B)** includes those patients with evidence of detectable ctDNA (red dot) and participant 1 (blue dot). If participant 1 is assumed to have ctDNA a strong positive linear relationship is seen between disease burden and cfDNA concentration in these patients (blue line of best fit shown) (R^2^=0.61). If participant 1 is assumed not to have ctDNA and removed from this analysis a weaker positive correlation between cfDNA levels and disease burden is seen (red line of best fit shown)(R^2^=0.34). According to RECIST 1.1 criteria patients 2, 4, 6, 8, 9 and 10 had progressive disease and patient 11 had stable disease when cfDNA samples were collected. Disease state could not be calculated for patients 1, 3, 5 and 7.

Total cfDNA concentration was significantly higher in the metastatic STS patient group than in the group of healthy controls analysed (48.37ng/ml (range 9.0-106.0) vs 3.9 ng/ml (range 1.9-7.4), P=0.006) (see Figure 1). A weakly positive linear relationship was present between disease burden and cfDNA concentration in the STS patients overall (R^2^=0.26), which increased in strength significantly when only those patients with evidence of ctDNA were analysed (R^2^=0.61) (see Figure 2).

### Formalin fixed paraffin embedded (FFPE) STS tissue DNA and circulating cfDNA sequencing

Genetic characterisation of circulating cfDNA, buffy coat (BC) DNA (representing normal germline DNA) and FFPE primary STS tissue DNA was conducted using a custom designed Ion AmpliSeq™ panel. Cell free DNA and BC DNA was available for all 11 enrolled patients (patients 1-11) whilst FFPE tumour tissue samples were available in 5 cases (patients 1-5.)

The analysis of samples from four patients (participants 2-5) provided clear evidence for the presence of ctDNA. In contrast the analysis of cfDNA from six other patients (participants 6-11) revealed no evidence of ctDNA. The analysis of samples collected from participant 1 revealed two polymorphisms at different frequencies in the STS DNA, BC DNA and cfDNA that can be interpreted in various ways. Only one of the potential explanations for the findings in this case supports the presence of ctDNA, making a definitive statement about presence or absence of ctDNA impossible. Detailed descriptions of the sequencing analyses of all these samples are shown below on an individual case basis.

#### Participant 1

Although the analysis of participant 1's FFPE STS DNA and cfDNA revealed no known cancer associated mutations, analysis of their BC DNA revealed two variant alleles at high frequencies. One was an intronic single nucleotide polymorphism (SNP) involving *TP53* (rs77697176, C/T) with a T allele read depth frequency of 31%, and the other a silent variant involving *HRAS* (rs12628_T/C_H27H) with a C allele frequency of 58%. The observed frequencies of the same variant *TP53* (rs77697176, T) and *HRAS* (rs12628, C) alleles in participant 1's STS DNA were 2% and 95% respectively (see Table 2). The frequencies of these *TP53* and *HRAS* alleles were 17% and 66% in participant 1's cfDNA. Taken together the pattern of allele frequencies can be explained in various ways.

**Table 2:** A summary of genetic changes identified in the STS patients analysed

							Patient cfDNA				Patient BC DNA			Patient Tumour DNA			Control cfDNA		
Pt	Chr	Position	Gene	Cosmic ID/dbSNP	Predicted effect	Variant Caller identified	Replicate data	Depth	Variant reads	%	Depth	Variant reads	%	Depth	Variant reads	%	Depth	Variant reads	%
1	11	534242	HRAS	COSM249860 rs12628	p.H27H Silent	Y	2527/3877 (65%) 1127/1654 (68%)	5531	3654	66	10660	6169	58	5395	5119	95	5202	13	0
	17	7576501	TP53	rs77697176	Intronic SNP	y	274/1482 (18%)73/575 (13%)	2057	347	17	2957	908	31	716	16	2	941	0	0
2	17	7577094	TP53	COSM10704	p.R282W Arginine◊tryptophan	y	257/5591 (5%)191/4457 (4%)	10048	448	4	2479	3	0	1307	1034	79	6071	4	0
3	3	178921495	PIK3CA	COSM1666843/4	p.S326F Serine◊Phenylalanine	n	\	6926	36	1	1898	2	0	2532	17	1	2075	4	0
4	3	178927410	PIK3CA	COSM328028	p.I391M Isoleucine◊Methionine	y	\	4008	110	3	2314	1	0	2139	1	0	3151	1	0
	17	7578210	TP53	COSM249885 rs1800372	p.R213R Silent	y	\	8114	293	4	2486	1	0	3438	3	0	4983	5	0
5	3	178916941	PIK3CA	COSM6145	p.E110K Glutamic acid◊Lysine	n	\	23716	148	1	10463	7	0	5506	17	0	1271	2	0

Data shown includes the sequencing analyses of patients’ cfDNA, BC DNA and FFPE tumour tissue DNA. The analysis of the cfDNA of one healthy control is also shown as a measure of background noise.

One interpretation assumes that participant 1 is germline heterozygous at both the *TP53* and *HRAS* SNPs, and that the observed allele frequencies in the tumour are the result of somatic loss of the T allele (LOH) at both sites. In this scenario, sampling error might explain the deviation from the expected 50% read depth for each allele seen in the BC (Table 2) although this observed deviation is higher than we witnessed in other SNPs during our analysis. If this interpretation is correct the presence of ctDNA may explain the deviance seen in the cfDNA allele frequencies away from their expected 50%, towards their tumoural frequencies (*TP53*, T, 17% and *HRAS*, C, 66%).

A second explanation for the altered tumour, BC and cfDNA allele frequencies is also based on the assumption that participant 1 is germline heterozygous at the *TP53* and *HRAS* SNPs, but that they are also mosaic for LOH in certain cell lineages following an early somatic event, including the haematopoietic progenitor cell population. In this scenario the deviation seen in the BC allele frequencies of the *TP53* and *HRAS* SNPs away from their expected 50% may be explained by a variable contribution to the circulating BC from the mosaic haematopoietic progenitor cell population. The presence of single alleles at each locus within the tumour could be explained by its origin from a cell with LOH at both loci. If this scenario is correct, the deviation seen in the cfDNA allele frequencies away from their expected 50% may be explained by either ctDNA or cfDNA shed from the mosaic haematopoietic BC population itself, precluding any statements on the presence of circulating tumoural DNA.

A third interpretation is that participant 1 was born a germline homozygote C at *TP53* (rs77697176) and *HRAS* (H27H, rs12628) but shows mosaicism at these loci for two independently acquired somatic C>T mutations in the patient's haematopoietic progenitor lineage [12]. The presence of these mosaic C>T mutations would explain the observed BC frequencies of the *TP53* rs77697176 (T/31%) and *HRAS* rs12628 (C/58%) polymorphisms. The contribution of participant 1's BC DNA to their cfDNA pool would also explain how the haematopoietic progenitor mosaicism could lead to the deviation seen in the cfDNA mutant allele frequencies away from those seen in the tumour (*TP53*_ rs77697176, T, 17% / *HRAS*_rs12628, C, 66%). This interpretation assumes that the tumour carries the homozygous germline genotypes at both loci (and therefore lacks the variant alleles generated by the somatic C>T mutations) and in terms of circulating nucleic acids provides no evidence for the presence of ctDNA.

#### Participant 2

A somatically acquired commonly reported cancer-associated *TP53* mutation (*TP53*_p.R282W_COSM10704) was detected in participant 2's FFPE tumour tissue and plasma at VAFs of 79% and 4% respectively.

#### Participant 3

Analysis of participant 3's FFPE tumour tissue DNA revealed a *PIK3CA* mutation (*PIK3CA_* p.S326F_ COSM16668843/4) at a VAF of 1%. Despite this low frequency, the same mutation was also detected in participant 3's plasma at a frequency of 1%.

#### Participant 4

No somatic mutations were detected in participant 4's FFPE STS DNA using our tNGS panel. Interestingly however two circulating *PIK3CA* mutations were detected in participant 4's cfDNA at VAFs of 3% (*PIK3CA*_p.I391M_COSM328028) and 4% (*TP53*_p.R213R_COSM249885) potentially highlighting the presence of significant intratumoural heterogeneity or clonal evolution.

#### Participant 5

Analysis of participant 5's FFPE STS tissue DNA revealed no somatic mutations. However, similarly to the scenario observed for participant 4, a circulating *PIK3CA* mutation (*PIK3CA*_p.E110K_COSM6145) was identified in participant 5's circulating cfDNA at a frequency of 1%.

#### Participants 6-11

No evidence of any circulating cancer associated variants (ctDNA) was identified in the plasma samples of participants 6-11. FFPE STS tissue for these participants was unavailable for analysis.

## DISCUSSION

STSs are a complex group of tumours with a relatively poor prognosis compared with many other solid cancers. One of the most significant barriers to improving STS patient outcome are the high levels of post-operative local or metastatic recurrence that follow primary treatment. The opportunity to offer radical, attempted curative treatment for this recurrence is dependent on early diagnosis, and as a result a lack of sensitive circulating STS biomarkers directly contributes to the poor prognosis of these recurrent patients.

The bloodstream contains several potential cancer biomarkers currently under investigation. These include circulating tumour cells (which enable single-cell sequencing as well as transcriptomic, proteomic and metabolomic analyses [13]), circulating extracellular microRNAs (which act as key regulators of gene expression and overcome the instability issues presented by other circulating RNAs [14]), and circulating nucleic acids.

Since cfDNA was first identified in cancer patients in 1973 [15] many potential clinical applications have been proposed and investigated in a translational setting. The analysis of cancer patient ctDNA is an attractive proposition for several reasons. These include ctDNA's ease of access, the diverse range of tumour characteristics that ctDNA analysis can provide, ctDNA's ability to overcome issues associated with Intratumoural Genetic Heterogeneity (IGH) and the high sensitivity and wide dynamic range through which ctDNA characteristics respond to changes in tumour behaviour. In addition to these advantages, the rapid clearance of cfDNA from the circulation (reportedly 30-120 minutes [16, 17]) means that the analysis of ctDNA provides an unparalleled reflection of a tumour's genomic makeup in real-time, assuming stringent protocols for plasma isolation are adhered to. Finally, the stabilisation that circulating nucleic acids undergo once removed from the circulation means that ctDNA can be stored and transported prior to analysis with no apparent detrimental effects on the quality of the resulting data [18].

The current body of literature describing ctDNA in STSs consists of only 2 case reports. The first of these used a case of metastatic intimal sarcoma to eloquently show how the analysis of ctDNA released from a patient's primary and metastatic tumours can be used to detect genomic heterogeneity between these lesions [10]. The second reported case evaluated the ctDNA characteristics of a patient with spindle cell sarcoma that developed disease recurrence soon after attempted curative treatment. Based on the detection of ctDNA both intra- and post-operatively, the authors of this study proposed that the persistence of microscopic disease post operatively explained this early relapse, and also highlighted a potential role for ctDNA as a marker of small volume disease in cases of STS [11].

To build on this limited body of work we initially compared overall cfDNA concentration in a group of metastatic STS patients with a group of healthy controls. As expected cfDNA levels were significantly higher in the STS patients that the healthy individuals. In addition cfDNA levels in the STS patients were shown to positively correlate with patient disease burden, but only fairly weakly (R^2^=0.26). In other malignancies the strength of this correlation varies [19, 20], and interestingly when those patients in our cohort with evidence of ctDNA were analysed separately, the strength of the correlation seen rose (see Figure 2) suggesting that a significant proportion of cfDNA in these cases was both tumour derived and released into the circulation in a linear correlation with disease burden. During the follow up period six of the 11 patients enrolled died of their disease. No significant difference was seen between the cfDNA levels of those patients that died and those that survived (P=0.34).

To look for evidence for ctDNA in the enrolled STS patients we next adopted a tNGS-based strategy. We created a custom Ion AmpliSeq™ panel specifically designed for use in STS patients which was used to sequence DNA extracted from a variety of samples collected from the enrolled STS patients. The panel was first used to analyse cfDNA collected from all 11 patients enrolled to look for circulating tumoural variants (and so ctDNA). Next, the same panel was used to analyse FFPE STS tissue DNA available for five of the enrolled patients to confirm the tumoural origin of any circulating variants identified. Finally the panel was used to sequence matched patient BC (germline) DNA to confirm the somatic origin of any circulating variants identified.

We found no definite evidence of ctDNA in the plasma of 7 of the patients analysed (participants 1, 6-11). This may reflect a true absence of ctDNA in these cases, suggesting that ctDNA is not released from every STS subtype, or indeed in every case of the same STS subtype. Alternatively it may be that ctDNA was present in these participants, but that it was not detected by the targeted custom Ion AmpliSeq™ panel used. This highlights a potential challenge with using ctDNA to monitor STSs, which is the absence of any point mutations found consistently throughout the group of tumours, in contrast to other malignancies such as melanoma and breast cancer [21, 22].

Among the 7 patients without evidence of ctDNA, only patient 1 suggested some ambiguity. Two polymorphisms were identified in this patient's STS, BC and cfDNA involving *TP53* (rs77697176) and *HRAS* (rs12628). As outlined above there are several potential explanations for the frequencies of the variant alleles in patient 1's tissues. The first is the presence of somatic LOH at both loci in the tumour, combined with significant sampling error and the presence of ctDNA. The second is the presence of somatically acquired LOH giving rise to mosaicism in the patient's haematopoietic lineage, as well as the tissues giving rise to the STS. The third is the presence of *de novo* somatic substitution mutations in clonal haematopoietic progenitor cell populations, which has recently been described as a contributory factor in cases of malignancy [12]. Of these scenarios only the first may indicate the tumoural shedding of DNA, and therefore based on the data presented it is not possible to conclude that ctDNA is present in this patient.

The analysis of patient 2's plasma revealed one known cancer associated mutation at a VAF of 4% (*TP53*_p.R282W_COSM10704). Analysis of patient 2’ FFPE STS tissue revealed the same mutation at high frequency (79%), providing strong evidence for the tumoural shedding of DNA into the circulation in this case. This is further supported by *TP53*_p.R282W's previously association with multiple mesenchymal tumours [23, 24], as well as early and late stage lung cancer patients where an association with progressive disease has been identified [25].

**Figure 3:**
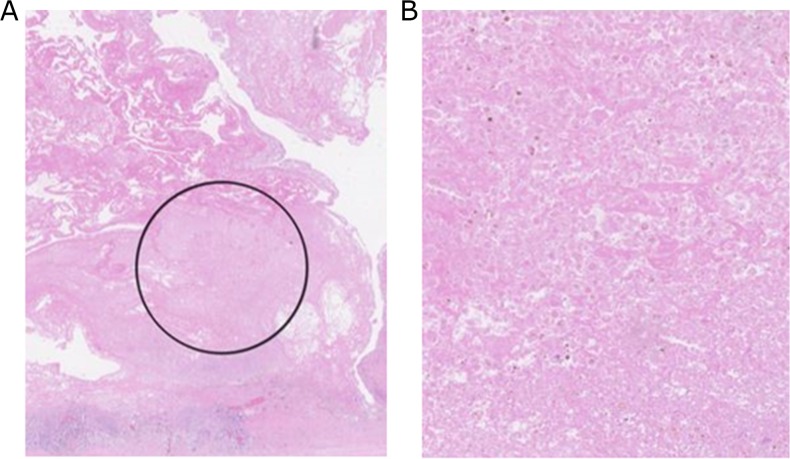
**(A)** H&E stained FFPE Undifferentiated pleomorphic sarcoma tissue from participant 3. The region highlighted by the black circle represents the area from which 1mm cores of tumour tissue were harvested for DNA extraction. **(B)** High power magnification of region circled in (A) from which DNA was extracted. Magnification and imaging was performed using a Hamamatsu slide scanner.

Analysis of patient 3's plasma revealed another circulating cancer associated mutation at a VAF of 1% (*PIK3CA*_p.S326F_COSM16668843/4). The same mutation was identified in patient 3's FFPE STS tissue providing strong evidence for the presence of ctDNA, particularly considering *PIK3CA_*p.S326F's previous associations with several malignancies [26]. The effects of dilution mean that a circulating variant's frequency is expected to be higher in its tumoural tissue of origin than in the plasma. Assuming all the FFPE cores analysed possessed a high tumour load (as suggested by H&E staining) (see Figure 3) we believe the equal VAF of *PIK3CA_*p.S326F in patient 3's plasma and FFPE tissue therefore reflects the presence of high frequency *PIK3CA_*p.S326F in tumour subclones not sampled in the FFPE cores analysed. This highlights another advantage of ctDNA analysis – its capacity to counter for intra-tumoural heterogeneity.

The analysis of patient 4 and 5's cfDNA revealed a total of three circulating variants absent from their FFPE STS tissue - *PIK3CA*_p.I391M_COSM328028 (participant 4), *TP53*_R213R_COSM249885 (participant 4) and *PIK3CA*_p.E110K_COSM6145 (participant 5). This finding may be explained in several ways. Firstly, the subclones containing these mutations within the patients’ STSs may not have been sampled during DNA extraction due to the presence of significant intra-tumoural heterogeneity. Secondly, the circulating mutations identified may have developed as a result of clonal evolution at some stage after these participants’ STSs were biopsied, prior to cfDNA collection (a period of time lasting up to 23 months). Thirdly, the circulating nucleic acids containing the variants may have been shed from mutated haematopoietic cells rather than tumoural cells. However, given role of *PIK3CA*_p.I391M and *PIK3CA*_ p.E110K in several malignancies [27–29], we consider this third option less likely (especially given the absence of both alterations in participant 4 and 5's sequenced BC) and propose that the variants’ presence in the circulation still provides strong evidence for ctDNA in these participants. The presence of circulating *TP53*_R213R in participant 4's plasma also highlights how somatic passenger mutations can act as useful tumour markers, even when synonymous and unlikely to have any oncogenic effect themselves.

A rational hypothesis would be that patients with the highest tumour burden and most aggressive STSs are most likely to have ctDNA in their plasma. This was not borne out by our data, with no significant difference seen between either the disease burden or the tumour grades of those patients with and without detectable ctDNA (P=0.71 and 0.52 respectively) (see Supplementary Figure 1) and no clear relationship evident between overall survival and the presence of ctDNA (see Table 1).

Overall our FFPE STS tissue analysis identified one cancer-associated mutation in 2 of the 5 cases analysed – one involving the tumour suppressor *TP53* and one involving the oncogene *PIK3CA*. This incidence of *TP53* and *PIK3CA* mutations is slightly higher than those previously reported in larger cohorts of STS patients (20% vs 17% and 20% vs 18% respectively [30]), although clearly this small difference is most likely a result of differences in the cohort sizes analysed (47 vs 11) and/or variation in the particular STS subtypes enrolled.

**Figure 4:**
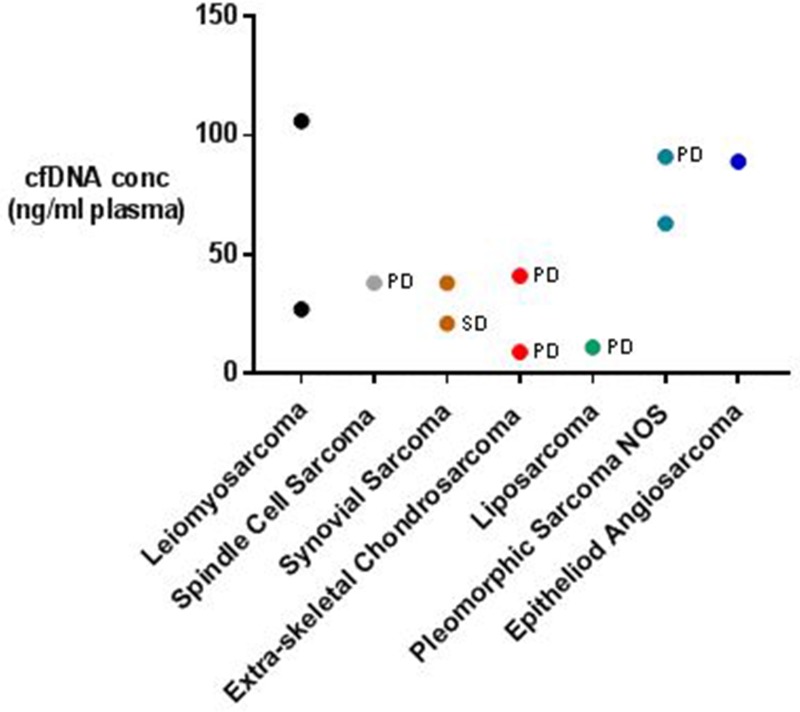
Metastatic STS patients’ cfDNA concentrations categorised according to STS subtype and disease state Disease state according to RECIST 1.1 criteria is recorded on the graph where available. PD = progressive disease, SD = stable disease.

Our aim was to characterise cfDNA/ctDNA in a cohort of multiple metastatic STS patients which we have successfully achieved. Our cfDNA quantification identified significantly elevated cfDNA levels in the STS patients analysed compared with healthy controls, as well as a positive correlation between disease burden and cfDNA concentration. By identifying these characteristics we have highlighted cfDNA levels as a potentially interestingly diagnostic or surveillance biomarker for late stage STS, but obviously further work is required to confirm this. In terms of ctDNA, our analysis has also successfully identified evidence of tumour derived circulating nucleic acids in 4 of the 11 patients in our cohort, including individuals with a wide variety of disease burdens. Although this has confirmed that ctDNA can be detected in cases of low volume stable STS, it also suggests that the targeted approach we adopted for the detection of ctDNA was insufficient to discriminate between the majority of the metastatic STS patients and healthy controls. This may reflect a low burden of simple point mutations or small indels in the STSs analysed or a need to increase assay sensitivity/specificity, but regardless necessitates further work before ctDNA can be volunteered as a source of novel STS biomarkers. Although our dataset builds significantly on the few previously published case reports on the topic, our sample size is small and does not allow us to make definitive statements about the relationship between ctDNA characteristics and STS grade, disease burden, patient prognosis, or the uniformity with which ctDNA is released from different STS subtypes (see Figure 4). Following this, future work should be designed to ensure that these relationships can be investigated independently. This work should also investigate patient cohorts in a longitudinal manner to allow correlation between circulating nucleic acid characteristics and key clinical outcome measures such as disease recurrence or progression – a necessity to determine the true potential of ctDNA analysis as a source of novel STS biomarkers.

## MATERIALS AND METHODS

### Ethics

This study was approved by the National Research Ethics Service Committee North East - Newcastle & North Tyneside (REC reference: 14/NE/1192) and the University of Leicester (project ref: nce8-cf5b4) and conducted in accordance with the Declaration of Helsinki (2013).

### Patients and samples

Eleven patients with a biopsy-proven metastatic STS were enrolled on a consecutive basis via the East Midland Sarcoma Service (United Kingdom). Consent was provided for the analysis of one 20ml sample of whole blood collected prospectively and where available the analysis of FFPE STS tissue blocks collected at the time of diagnosis (available for participants 1-5). Whole blood was also collected from 6 healthy participants (2m: 4f) with a mean age of 45 (32-59) for cfDNA concentration comparison. Circulating DNA from one of these participants was sequenced using our tNGS panel as internal quality control to ensure the panel performance on cfDNA templates and to identify and exclude any sequencing artefacts. All blood samples were processed within 2 hours of collection to isolate plasma cfDNA and BC DNA as previously described [31]. Prior to enrolment, each STS patient's biopsy specimens and radiological investigations were reviewed by a consultant histopathologist and radiologist with a specialist interest in soft tissue tumours to confirm eligibility.

### Disease burden

To objectively gauge disease burden, each patient's most recent cross-sectional radiological investigations were reviewed by a consultant radiologist with a specialist interest in musculoskeletal tumours and scored according to RECIST 1.1 criteria. RECIST scores were defined as the sum of all target lesions’ diameters. Where serial comparable imaging was available a measure of disease state was also calculated in accordance with the same RECIST 1.1 criteria. Progressive disease was defined as a 20% increase in the sum of measurable lesions’ diameters between consecutive scans.

### Adjuvant therapy

Clinical notes were reviewed to confirm STS subtype and Trojani tumour grade where available/applicable. Hospital records were also reviewed to ascertain if any systemic medical therapies or radiotherapy was given prior to sample collection.

### DNA extraction

Tumour DNA was extracted from 1mm FFPE tissue cores using the FFPE Gene Read Kit (Qiagen). Prior to DNA extraction all FFPE blocks were reviewed by a histopathologist to ensure tissue was not taken from necrotic or misrepresentative regions. Circulating cfDNA was extracted from plasma samples using the QIAamp Circulating Nucleic Acid Kit (Qiagen). Buffy coat DNA was extracted using the QIAamp DNA Mini Kit (Qiagen). All kits were used according to the manufacturer's protocols.

### DNA quantification

Circulating cfDNA yields were determined using High Sensitivity D5000 ScreenTapes on a 4200 TapeStation Instrument (Agilent Technologies) and quantitative PCR. Buffy coat and tumour DNA was quantified using a Qubit® 2.0 Fluorometer or a Nanodrop spectrophotometer (Thermo Fisher Scientific).

### Targeted next generation sequencing (tNGS)

To identify tumour-derived somatic mutations we created a custom Ion AmpliSeq™ panel designed to target 57 regions either previously reported in STSs or associated with the pathological maintenance of telomere length (a necessity for all malignant cells to achieve replicative immortality). The panel was designed following a detailed review of multiple online databases of large genomic studies (http://cancer.sanger.ac.uk/cosmic/ www.cbioportal.org) and included a total of 9600 COSMIC registered mutations (see Supplementary Table 1/2 for full panel description). DNA libraries were prepared using the Ion AmpliSeq™ Library Kit v2.0 (ThermoFisher) according to manufacturer's instructions and sequencing was performed on an IonTorrent PGM. This panel was used to sequence cfDNA collected from all 11 enrolled patients where the presence of ≥1 somatic variant registered on the Catalogue of Somatic Mutations in Cancer database (http://cancer.sanger.ac.uk/cosmic) was assumed to be representative of ctDNA. The same panel was next used to sequence matched leucocyte-derived BC DNA to confirm the somatic origin of any low frequency mutations identified, and in 5 of the 11 enrolled STS patients FFPE STS DNA to confirm the tumoural origin of any circulating variants identified. Ten nanograms of template DNA was used for each reaction and where cfDNA yields allowed, cfDNA was sequenced in duplicates.

### Variant calling

Sequencing data was aligned against hg19. Somatic variants were identified using a two-step approach: First, sequencing data was analysed using the Variant Caller software instructed to detect low frequency variants present at more than 1% of the sequence reads. A hotspot file was generated to cover the COSMIC positions included in the panel to increase the variant call sensitivity at these positions to 0.2%. Next, every amplicon amplified by our panel was manually reviewed in each patient using the Integrated Genomics Viewer (IGV) package (v2.3.25) to identify any variants missed by the Variant Caller software. All of the variants identified by the Variant Caller were also manually inspected to rule out the presence of sequencing artefacts.

### Statistical analysis

Statistical analysis was performed using GraphPad Prism 7.0. Data was assumed to have a normal distribution and compared using unpaired *T*-test and Pearson's correlation.

## SUPPLEMENTARY MATERIALS FIGURE AND TABLES







## Abbreviations

tNGS: Targeted next generation sequencing
STSs: Soft tissue sarcomas (STSs)
ctDNA: Circulating tumour-derived DNA
cfDNA: Cell free DNA
TP53: Tumour protein 53
PIK3CA: Phosphatidylinositol-4, 5-bisphosphate 3-kinase catalytic subunit alpha
FFPE: Formalin fixed paraffin embedded
BC: Buffy coat
VAFs: Variant allele frequencies
IGH: Intratumoral Genetic Heterogeneity
LOH: Loss of heterozygosity
