# Supplementary material for: Circulating tumour-derived DNA in metastatic soft tissue sarcoma
Source: Oncotarget. 2018 Jan 19;9(12):10549–60. doi: 10.18632/oncotarget.24278 (PMC5828212; doi:10.18632/oncotarget.24278)
Supplement: Supplementary file 1 [file oncotarget-09-10549-s001.pdf]

Circulating tumour-derived DNA in metastatic soft tissue sarcoma

SUPPLEMENTARY MATERIALS

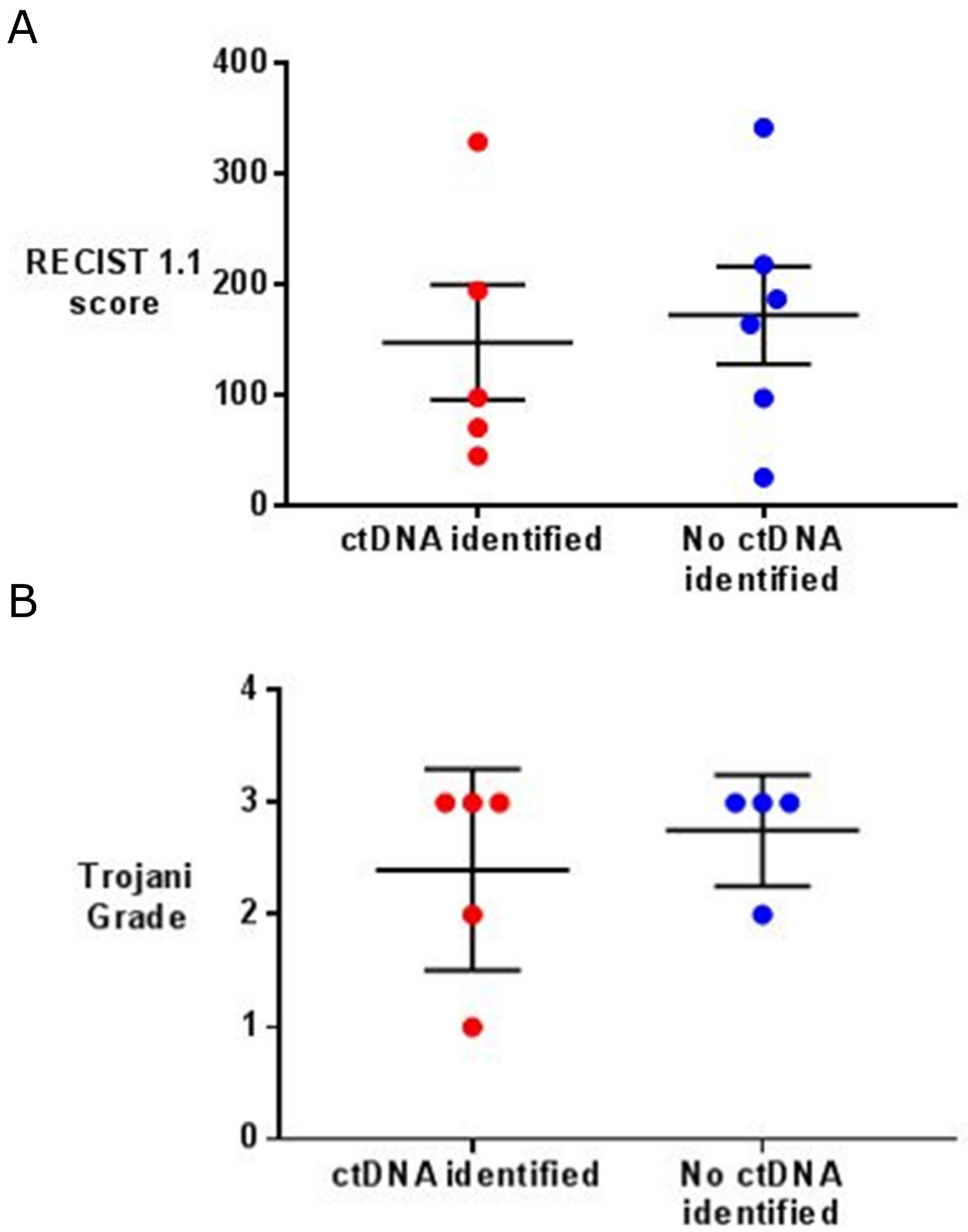

Supplementary Figure 1: Graphs comparing the disease characteristics of metastatic STS patients with and without evidence of ctDNA. Comparisons between the groups' disease burdens (RECIST 1.1 score) and Trojani tumour grades are shown in **(A and B)** respectively. Horizontal bars represent mean values and standard error of the mean. No significant difference was seen between groups (P=0.72 and 0.51 respectively, unpaired *t*-test).

Supplementary Table 1: List of designed Ampliseq^{TM} panel's amplicons' start and end positons including chromosome and target genes.

See Supplementary File 1

Supplementary Table 2: List of hotspot locations and COSMIC ID registered mutations covered by the designed Ampliseq^{TM} panel including chromosomes and base changes involved.

See Supplementary File 2
